# Tonsillitis Caused by Vomiting in a Patient with Bulimia Nervosa: A Case Report and Literature Review

**DOI:** 10.1155/2013/251629

**Published:** 2013-03-03

**Authors:** Miles Bannister

**Affiliations:** ENT Department, Tameside General Hospital, Fountain Street, Ashton-under-Lyne OL6 9RW, UK

## Abstract

A 32-year-old lady presented to our ENT service with worsening tonsillitis. This was one of multiple attacks; all of which had all followed periods of self-induced vomiting due to the patient suffering with bulimia nervosa. Here we present the first ever case report of such a case of tonsillitis and a review of the literature of otolaryngology manifestations and complications of bulimia nervosa.

## 1. Introduction

Bulimia nervosa is a psychological disorder involving cyclical purging following eating. Otolaryngological manifestations centre on sialomegaly, oral disease, and the complications of self-induced purging. We discuss a case of vomiting-induced tonsillitis in a bulimia nervosa sufferer and review the published literature of otolaryngology associations with bulimia nervosa.

## 2. Case Presentation

A 32-year-old lady presented to our emergency ENT service complaining of a sore throat. This had developed over a 7-day period and was associated with a fever and odynophagia of solids and liquids. Her symptoms had failed to improve despite a four-day course of benzylpenicillin 500 mg qds and a two-day course of metronidazole 400 mg tds. The patient reported no past medical history but revealed that she had suffered from bulimia for 15 years although general practitioner review had declined further contact with the psychiatric services because of difficult previous experiences with them. She described fortnightly purging following eating during the previous 6 months, which was followed by episodes of tonsillitis. She used no regular medications. ENT examination revealed bilaterally enlarged, erythematous tonsils, and jugulodigastric lymphadenopathy. No peri-tonsillar abscess was present. Ear examination and nose examination were normal. Blood testing was normal throughout. IgM screen was negative. The patient was admitted to our ENT service for intravenous antibiotic therapy. The patient declined followup for consideration of a tonsillectomy as she was moving to a different region. The patient was advised to contact her new local otolaryngology service for tonsillectomy consideration.

## 3. Discussion

Bulimia nervosa is a psychological eating disorder involving cyclical purging following eating. Up to 95% of sufferers are women [1]. The peak incidence is in young women [1]. The disorder was formally identified in 1979, although a previous case had been reported as early as the nineteenth century [2, 3]. Cyclical purging following eating was commonplace in Roman civilisation.

Salivary gland enlargement was the earliest reported otolaryngology manifestation of bulimia nervosa. By 1986, 6 separate cases of sialomegaly had been reported [4]. Three of these cases were reported before bulimia nervosa became formally recognised [4]. Sialomegaly can manifest in 10–50% of bulimics [5, 6]. The parotid gland is the commonest affected gland with sialomegaly being diffuse, bilateral, and painless [5]. Salivary gland hypertrophy may be due to an increases in salivary demand following abrupt stimulation or as a result of pancreatic damage [6]. Parotid duct irritation from vomit may be another cause [6]. Salivary gland biopsy displays no histological features of inflammation [6]. The saliva flow rate from the glands may reduce whilst salivary amylase levels may rise [6]. Different surgeons have advocated the benefits and disadvantages of parotidectomy for bulimia-induced parotomegaly [7, 8]. A balance should be struck between any psychological benefits from surgery against the complications of parotidectomy. Sialomegaly will rescind with bulimia cessation [5].

Oral disease may result from repeated vomiting. Dental enamel made erode, particularly on the lingual surface of the maxillary teeth [4]. Enamel erosion of the incisors may close the jaw bite because the clinical crowns have shortened [4]. Enamel loss also sensitises the sufferer to hot foods and liquids [4]. Teeth may dislodge following damage from vomiting. Rothstein and Rothstein reported a case of a displaced tooth being swallowed by a patient, which presented as mid-sternal pain on swallowing [9]. Hands and fingers may develop calluses from friction generated by rubbing against the teeth during attempts to induce vomiting [4]. Erythema of the oral cavity and cheilosis may be presenting signs of bulimia nervosa, as can skin erosion and cracking over the lips [4]. Cheilosis describes fissuring at the angles of the mouth and is similar to angular stomatitis [4].

Oesophageal obstruction has been reported in bulimia nervosa sufferers. Sastry et al. reported a case of a metal teaspoon that was used to induce vomiting, being accidentally swallowed in a bulimia sufferer [10]. The teaspoon was removed through rigid oesophagoscopy and gastrografin swallow imaging was used to assess for any resulting oesophageal perforation. Rothstein and Rothstein reported an earlier case of undigested food obstructing the oesophagus following attempted vomiting [9]. The patient presented with dysphagia and flexible laryngoscopy revealed secretion pooling in the pyriform fossa. The obstructing food bolus was also removed through rigid oesophagoscopy. Barium swallow imaging following removal was satisfactory.

Tissue injury due to attempts at self-induced vomiting may present to an ENT service. Delap et al. reported a case of retropharyngeal abscess development following posterior pharyngeal wall injury from a fork used by a sufferer to induce vomiting [11]. The abscess that developed extended from the skull base to the posterior mediastinum. The condition was managed by incision and drainage and intravenous antibiotics. S. G. Rothstein and J. M. Rothstein report a less serious case of pharyngeal wall haemorrhage following trauma with a spoon that was used to induce vomiting by a bulimia sufferer [9]. The haemorrhage was arrested with silver nitrate cautery with no further complications.

## 4. Conclusion

 We report a new otolaryngological manifestation of bulimia nervosa and a new aetiological cause of recurrent tonsillitis. Otolaryngologists should be aware that recurrent tonsillitis may be a manifestation of bulimia nervosa in those groups in whom the disorder is most prevalent, as well as other manifestations of bulimia nervosa. General practitioners and psychiatrists should be aware of the head and neck complications of bulimia nervosa too. The management of otolaryngology complications should not alter because of the concurrent history of bulimia nervosa.

## References

[1] Pyle RL, Halvorson PA, Neuman PA, Mitchell JE (1986). The increasing prevalence of bulimia in freshman college students. *International Journal of Eating Disorders*.

[2] Russell GF (1979). Bulimia nervosa: an ominous variant of anorexia nervosa. *Psychological Medicine*.

[3] Leo H (1889). Uber bulimic. *Deutsche Medizinische Wochenschrift*.

[4] Abrams RA, Ruff JC (1986). Oral signs and symptoms in the diagnosis of bulimia. *The Journal of the American Dental Association*.

[5] Vavrina J, Muller W, Gebbers J-O (1994). Enlargement of salivary glands in bulimia. *The Journal of Laryngology and Otology*.

[6] Riad M, Barton JR, Wilson JA, Freeman CPL, Maran AGD (1991). Parotid salivary secretory pattern in bulimia nervosa. *Acta Oto-Laryngologica*.

[7] Berke GS, Calcaterra TC (1985). Parotid hypertrophy with bulimia: a report of surgical management. *Laryngoscope*.

[8] Rauch SD, Herzog DB (1987). Parotidectomy for bulimia: a dissenting view. *American Journal of Otolaryngology*.

[9] Rothstein SG, Rothstein JM (1992). Bulimia: the otolaryngology head and neck perspective. *Ear, Nose and Throat Journal*.

[10] Sastry A, Karkos PD, Leong S, Hampal S (2008). Bulimia and oesophageal foreign bodies. *The Journal of Laryngology and Otology*.

[11] Delap TG, Grant WE, Dick R (1996). Retropharyngeal abscess-an unusual complication of anorexia nervosa. *The Journal of Laryngology and Otology*.

